# Effects of chewing on postural learning: An experimental pre-post intervention study

**DOI:** 10.1371/journal.pone.0330355

**Published:** 2025-09-04

**Authors:** Cristina Dolciotti, Paolo Andre, Maria Paola Tramonti Fantozzi, Francesco Lazzerini, Vincenzo De Cicco, Massimo Barresi, Claudia Grasso, Luca Bruschini, Davide De Cicco, Paolo Orsini, Francesco Montanari, Ugo Faraguna, Diego Manzoni

**Affiliations:** 1 Department of Translational Research and of New Surgical and Medical Technologies, University of Pisa, Pisa, Italy; 2 Department of Medicine, Surgery and Neuroscience, University of Siena, Siena, Italy; 3 Department of Surgical, Medical and Molecular Pathology and Critical Care Medicine, University of Pisa, Pisa, Italy; 4 Maxillofacial Surgery Unit, Italian Stomatology Institute, Milan, Italy; 5 Department of Developmental Neuroscience, IRCCS Fondazione Stella Maris, Pisa, Italy; University of Innsbruck, AUSTRIA

## Abstract

In 16 healthy volunteers (age 42–69 years, 8 females) we investigated chewing effects on postural learning. Initially, the Centre of Pressure (CoP) position in bipedal stance was recorded (1 minute) in 4 conditions: Hard support (HS)-Open Eyes (OE), HS-Closed Eyes (CE), Soft Support (SS)-OE, SS-CE. Following 2 minutes of Chewing (C, n = 8 subjects, 4 females) or rhythmic Hand Grip (HG, n = 8 subjects, 4 females), 10 unipedal stance test (1 minute) were performed for 30 minutes in both groups in HS-OE, with a progressive decrease in CoP Velocity and Path Length. Since the 95% Area of body sway decreased only in the HG group, the Length in Function of Surface (LFS, indicative of balance energy expenditure), increased in the HG and remained constant in the C group. Soon after and 5 hours post-training, bipedal stance tests were performed for 8 minutes, in the same order as before. In both groups, the changes in unipedal stance parameters were found persistent 5 hours post-training. In SS-OE condition of bipedal stance, CoP Velocity was reduced and 95% Area increased by postural training, in the HG and C group, respectively. These modifications were significantly correlated to the corresponding changes in unipedal stance and led to a LSF decrease in the C group. In conclusion, the CoP Velocity during unipedal training was not affected by the previous motor activities. Chewing allowed for a larger compliance concerning the extent of CoP oscillation. Postural training in unipedal stance seem to favour the development of modifications in bipedal stance, according to the conditioning activity. Chewing before a postural training promotes a postural strategy characterized by a constant and a lower energy cost in unipedal and bipedal stance, respectively. Further experiments are necessary to verify whether such a change may promote a more secure balance in trained people.

## Introduction

### Trigeminal input improves cognitive functions

Chewing improves overall health and cognitive functions. Poor chewing is linked to cognitive decline, while efficient chewing improves alertness [1,2], attention [3], memory and learning [4–6], task performance [7,8], cerebral blood flow [9] and mydriasis [7,8], accelerates cognitive processing [10] and reduces reaction times and latencies of Event Related Potentials (ERPs) [11], unlike mere jaw or hand rhythmic movements [7].

### The Locus Coeruleus links trigeminal input and cognitive functions

It has been suggested that sensorimotor trigeminal signals during chewing enhance the activity of the Ascending Reticular Activating System (ARAS), boosting arousal [12]. Indeed, in the *encephale isolé* preparation, trigeminal nerve lesions cause persistent electroencephalographic synchronization [13]. Notably, noradrenergic neurons in the Locus Coeruleus (LC) are strongly activated by trigeminal stimulation [14], through several pathways [12], including direct projections from the trigeminal mesencephalic nucleus (Me5) [15,16]. Additionally, improved cognition from chewing aligns with increased pupil dilatation during the task. Since pupil size is a proxy of the LC activity [17–20], this finding indicates enhanced phasic LC activation [7], which plays a crucial role in arousal and cognitive performance [21,22]. While chewing stimulates cognitive performance, the presence of a right-left asymmetry in mandible elevator electromyographic (EMG) activity during clenching impairs it [23,24]. This trigeminal asymmetry correlates with the pupil size asymmetry (anisocoria) observed with the masticatory muscles relaxed. Thus, the impairment is related to an asymmetric LC discharge at rest.

### Trigeminal influence on posture and movement

There is substantial evidence regarding the positive effects of trigeminal sensorimotor activity on cognitive functions, whereas less is presently known about its influence on body posture and movement. Both humans and animals can use the stomatognathic system for interacting with the environment. It is therefore reasonable to assume that orofacial inputs elicited by this interaction may affect postural muscles, in the same way as it occurs when these signal arise from the use of the upper limb [25–27]. Moreover, both in humans and animals, trigeminal afferents affect the activity of neck muscles, leading to a head withdrawal reflex [28,29], while trigeminal stimulation drives the complex crawling movements that allow immature marsupials to reach the maternal nipple within the pouch [30]. In apparently normal subjects, placing silicon disks between the arches alters the position of the vertebral column [31], whereas the use of bite splint [32] makes locomotion more symmetric. During clenching, trigeminal afferents contribute to the concurrent enhancement of soleus motoneurons excitability [33], leading to a greater co-contraction of agonist and antagonist muscles and minimizing joint motion [34] and the Centre of Pressure (CoP) excursion during bipedal stance [35]. Finally, increased postural stability is observed during chewing in healthy individuals [36,37]. These findings can be related to the direct connections of trigeminal afferents with spinal circuits [38,39] (see however [40]), as well as to trigemino-spinal pathways running through the trigeminal nuclei [41,42], the vestibular nuclei [43,44] and the reticular formation [45].

Despite these anatomic, functional and behavioural data, there is no agreement about the possible impact of a disordered trigeminal input on body muscles and postural function [46–48]. Occlusal correction in individuals with Temporo-Mandibular Joint dysfunction [49] does not impact visual reaction time and movement time, muscular strength, submaximal and maximal oxygen uptake, aerobic/anaerobic power output, and all-out working capacity in both arm and leg exercise [50]. At variance, in subjects with asymmetric masseter EMG during clenching (trigeminal imbalance), occlusal correction reduced the activation in sensorimotor cortical regions and in the cerebellum during a finger-to-thumb motor task [51], suggesting that less effort is required for movement control/programming when the trigeminal imbalance is reduced [51]. Moreover, in edentulous patients, a complete denture [52] improves postural stability at rest and during locomotion, while, in subjects affected by various forms of malocclusion, cotton rolls interposition between the arches enhances postural control on an unstable support [53]. Finally, increased postural stability is observed during chewing in patients with equilibrium disorders [54].

So far, the acute influence of stomatognathic information on motor learning have not been addressed. The present investigation addresses the effects of chewing activity on postural learning, which is relevant in sports training and rehabilitation practices [55–59] and enhances balance when support [55] or position (i.e., unipedal stance [55,60]) are unstable.

Postural learning mechanisms are regulated by the cerebellar vermis [61–65] and by the noradrenergic fibres impinging on this region [66,67], which arise from the LC [68–70]. Since trigeminal input affects LC activity and the LC influences the cerebellar vermis, it could be proposed that chewing can improve postural learning. Thus, we compared the changes in CoP motion during unipedal stance training in two groups of participants. One group chewed for 2 minutes before postural training (Chewing, C), while the other performed a rhythmic handgrip exercise (Handgrip, HG). The postural changes and their retention 5 hours post-training were compared between groups.

## Materials and methods

### Subjects

Participants were screened through a preliminary interview conducted by the research team, during which they were asked about symptoms related to cardiovascular, respiratory, metabolic, neurological, vestibular, psychiatric, and musculoskeletal conditions. Individuals with a history of orthopaedic prostheses or spinal arthrodesis were excluded. Additional exclusion criteria included the presence of severe cardiovascular disease (e.g., heart failure, complex arrhythmias, recent ischemic events), active neurological or psychiatric disorders, permanent sequelae from prior cerebrovascular events, chronic-progressive neurodegenerative or autoimmune diseases (e.g., Parkinson’s disease, multiple sclerosis), severe uncorrectable visual impairment, recurrent vestibular symptoms, and severe metabolic or respiratory conditions in an active phase. In addition to the clinical interview, participants underwent a structured screening to evaluate cognitive, balance, and vestibular functions. Cognitive performance was assessed using the Mini-Mental State Examination (MMSE, [71]), with a score of 24/30 or higher required for inclusion. Static and dynamic balance were evaluated using the Berg Balance Scale (BBS, [72]), with a minimum required score of 46/56. Vestibular function was assessed using the Fukuda stepping test [73]:participants exhibiting a lateral rotation greater than 30 degrees to either side were excluded. The hand dominance was assessed by the Waterloo Handedness Questionnaire [74].

**Fig 1:**
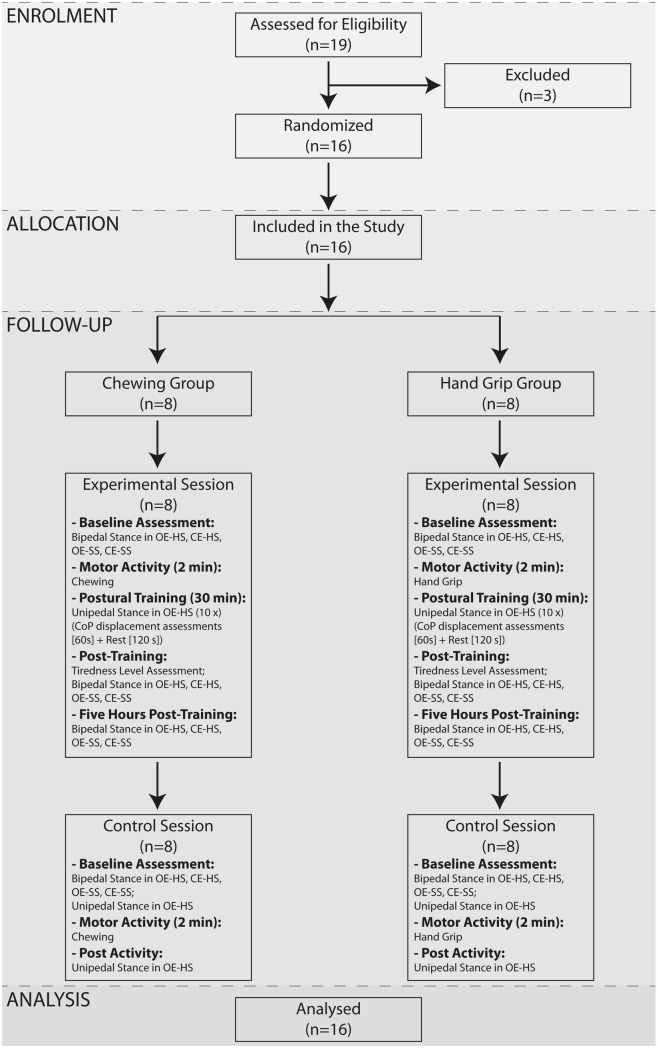
Flow diagram. Flow-chart showing subject enrolment, randomization and their progression through the study. CoP: Centre of Pressure. OE: Open Eyes. CE: Closed Eyes. SS: Soft Support. HS: Hard Support.

Following this screening, 16 healthy, right-handed volunteers (females: n = 8, age 55.1 ± 5.6 years; males: n = 8, age 52.6 ± 8.0 years) were enrolled in the experiment from an initial population of 19 subjects (Fig 1). All the subjects had handedness and footedness scores largely positive, with average values of 61.0 ± 7.9 and 15.9 ± 2.7, respectively. The study was approved by the Ethics Committee of the University of Pisa (N. 28/2022) and all the participants signed a written informed consent. Subjects’ recruitment was performed between 31/5/2023 and 10/10/2024.

### Experimental design

Participants were pseudo randomly assigned to the two experimental groups: Chewing (C – females: n = 4, age 54.3 ± 5.0 years; males: n = 4, age 54.5 ± 9.6 years) or Hand Grip (HG – females: n = 4, age 56.0 ± 6.0 years; males: n = 4, age 50.8 ± 5.3 years) groups. No significant difference could be observed between the two groups for height (C: 1.74 ± 0.08 m, HG: 1.70 ± 0.08 m) and body weight (C: 70.88 ± 10.15 Kg, HG: 67.63 ± 7.76 Kg). The experimental design (Fig 1) included two different sessions, the training (experimental) and the control sessions, spaced by at least three months, which begun in the morning.

The training session started at about 10 a.m., with an evaluation of CoP displacement of subjects standing barefoot on a stabilometric platform in bipedal stance. Before each recording, subjects were fixating a target at eyes level, 90 cm away. Stabilometric signals were acquired with eyes open (EO) and closed (EC), on hard (HS) and soft support (SS), according to the following sequence: OE-HS, OE-SS, CE-HS, CE-SS. Subjects were allowed to rest for 120 sec between conditions. Then the subjects of the C group received a chewing gum pellet and were invited to chew it on one side of his/her choice. After 60 sec subjects had to chew a second pellet on the other side for another 60 sec. Subjects in the HG group were asked to perform a rhythmic hand grip exercise (rhythmic closure of the fist at the subject’s spontaneous rate) first with the dominant hand (60 sec) and then with the other (60 sec). Soon after, both groups started a postural training consisting of 10 consecutive, 60 sec assessments of CoP displacement in unipedal stance on their preferred leg, in OE-HS conditions. Each recording was followed by 2 minutes sitting rest, for a total time of 30 minutes including 10 measurements (0–3, 3–6, 6–9, 9–12, 12–15, 15–18, 18–21, 21–24, 24–27 and 27–30 min time points). The whole sequence of CoP displacement recordings in bipedal stance was repeated soon after the postural training: overall, this initial part of the session lasted about 50–60 minutes. Five hours after the end of training, all bipedal stance recordings were performed once more, together with an additional testing in unipedal stance.

During unipedal stance, occasional loss of equilibrium occurred, obliging the subjects to transiently use both leg for stance. The number of these episodes was annotated by the experimenter. Finally, at the end of the training period, the subjects were asked to rate their level of tiredness on a visual analogue scale spanning from 0 to 10 [75].

In the control session, participants belonging to both groups were submitted to the whole sequence of CoP measurements performed in bipedal stance during the experimental session and to an evaluation in unipedal stance. Following a period of the group specific motor activity (2 min), they were tested once more in unipedal stance (Fig 1).

### Pellets

The chewed pellets were made by ordinary gum base (46.75%), isomalt (31.9%), luteolin (11.0%) and mannitol (9.0%). Small amounts of magnesium stearate, silicon dioxide  water and talc were also present. Their initial hardness corresponded to 60 Shore.

### Stomatognathic and pupillometric evaluation

In a separate session, the masseter EMG activity was evaluated in all subjects during clenching, and pupil size was measured while elevator muscles were relaxed, and the arches were slightly apart or in contact. Masseter EMG activity during clenching was recorded by Duo-trode surface Ag/AgCl electrodes (inter-electrode distance 19.5 mm, Myo Tronics, Seattle, WA, USA). Details about electrode placement have been reported in a previous study [23]. The EMG activity was sampled at 720 Hz by a K6-I Myo Tronics system, high-pass (cutoff frequency 15 Hz) and notch (50 Hz) filtered, full-wave rectified and displayed on the instruments monitor, together with the mean value of the rectified EMG bursts.

Pupil size was measured by a corneal topographer-pupillographer (MOD i02, with chin support, CSO, Florence, Italy), whose halogen lamp (white light) provided a constant artificial lighting of 40 lux (photopic condition). The instrument, endowed with a camera sensor CCD1/3” (working distance: 56 mm), allowed to monitor the iris image with an acquisition time of 33 msec; measurements performed for both eyes were immediately displayed on the computer screen and the pupil images were stored on disk. Measurements were performed 33 msec after turning off the instrument lamp before pupil dilatation began (about 300–635 ms, [76,77]).

Asymmetries in EMG activity and pupil size (anisocoria) were evaluated as percentage of the left-right difference with respect to the left-right average value.

### CoP displacement recordings

The position of the CoP was recorded through a stabilometric platform (Dune 2000; Dune S.A.R.L., Negenheim, France), endowed with three force sensors disposed at the apexes of an equilateral triangle with one of the sides parallel to the platform posterior edge. The origin of the coordinates system for CoP monitoring was placed within the centre of the triangle, at about the midpoint of the inter-feet line. Signals from the force sensors were separately acquired and analysed through a Lab-view software prepared ad hoc (sampling rate: 2KHz). This software calculated the time course of X and Y coordinates of the CoP starting from the output of platform’s force sensors. The obtained CoP traces were submitted to a smoothing procedure in which a given point (at the N^th^ position in the trace) was substituted by the average of all (101) the points included between N+50 and N-50. This procedure did not modify the time course of the traces.

The postural parameters provided by the program were: X and Y Sway Amplitude (standard deviation of CoP position for X and Y axes), 95% Area (area of the 95% confidence ellipse), Path Length (length of the CoP path during the 60 sec of the recording period), Length in Function of Surface (LFS: Path Length/95% Area) and Velocity (Path Length/60 sec). When a subject underwent a loss of equilibrium in unipedal stance, the corresponding portions of the CoP position traces were disregarded, and the duration of the analysed frames was shorter than 60 sec. In these instances, the Path Length was divided by the duration of the analysed frame and multiplied by 60, so to make these measurements comparable to those obtained for complete 60 sec trials.

### Statistical analysis

Data relative to unipedal stance were averaged over 6 min time windows (each including two time points) and analysed by a 5 Time (0–6, 6–12, 12–18,18–24, 24–30 min) repeated measure ANOVA with Group (C, HG) as between-subjects factor.

Possible modifications observed in bipedal stance parameters following postural training were analysed by a 3 Time (initial, end-training, 5 h post-training) repeated measures ANOVA, with Group (C, HG) as between-subjects factor. The analysis was applied separately to each of the 4 conditions of stance (OE-HS, OE-SS, CE-HS, CE-SS). Tiredness scores were compared between groups by independent t test.

In addition, when Group effects or interactions were found, we also separately evaluated, for each group, average parameters values of males and females at different time points, which were compared by independent t test to detect significant sex differences.

Finally, a linear regression model was utilised to study the correlations between variables. This analysis was used for comparing (unipedal and bipedal) stance data obtained in the training and in the control session. Moreover, the changes in unipedal stance data were expressed as a difference with respect to 0–6 min time point and correlated with this initial value. These data were also utilized to study the coupling between the changes observed in the different parameters of unipedal stance. Finally, the differences between critical time points (end-training/initial, 5h post-training/initial, 5h post-training/end-training) were evaluated in both groups and correlated with each other to assess whether training-induced changes in unipedal and bipedal stance were associated with each other.

All the statistical computations were performed by a SPSS software package (Statistical Package for Social Sciences, version 20, IBM). Correction for non-sphericity was applied to ANOVA when necessary. Contrast analysis was used between conditions. The significance level was set at p = 0.05

## Results

### Training-induced changes in unipedal stance

**Fig 2:**
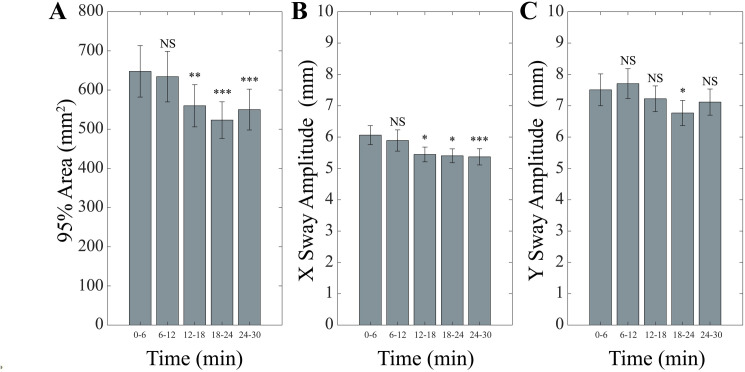
Time course of 95% Area, X and Y Amplitude Sway during postural training. Changes in 95% Area (A), X (B) and Y (C) Sway Amplitude observed in unipedal stance at the different time intervals of postural training. Error bars correspond to SE. Each time point has been compared to the initial (0-6 min) one. NS: not significant. *: p < 0.05. **: p < 0.01. ***: p < 0.005.

**Fig 3:**
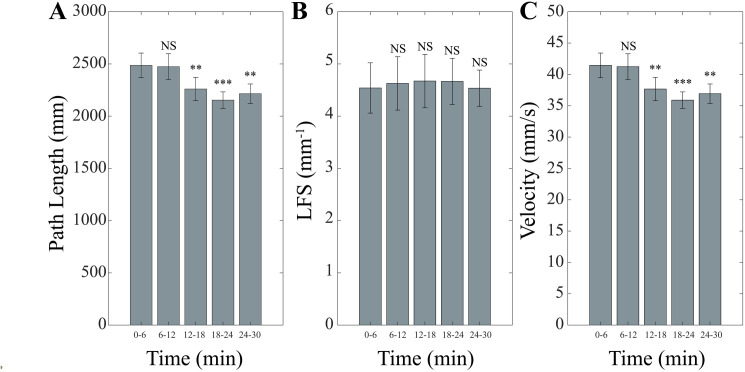
Time course of Path Length, LFS and Velocity during postural training. Changes in Path Length (A), LFS (B) and Velocity (C) observed in unipedal stance at the different time intervals of postural training. Error bars correspond to SE. Each time point has been compared to the initial (0-6 min) one. NS: not significant. *: p < 0.05. **: p < 0.01. ***: p < 0.005.

The evolution of unipedal stance parameters during the 30 min period of postural training was analysed by a 5 Time repeated measure ANOVA with Group as a between-subjects factor. Significant Time effects were observed for 95% Area (F(4,56)=7.19, p < 0.0005), Y Sway Amplitude (F(4,56)=4.10, p = 0.006), X Sway Amplitude (F(4,56)=5.299, p = 0.001, Path Length (F(4,56)=7.68, p < 0.0005) and Velocity (F(4,56)=7.68, p < 0.0005) but not for LFS. In all instances the values decreased significantly from time 6–24 and levelled at 30 min (Figs 2 and 3). Average values for each parameter at the different time points of the training session are given in Table 1.

**Table 1:** Mean ± SD values of unipedal stance parameters evaluated at the different time points of the postural training session.

	A. 0-6 min	B. 6-12 min	B vs A (p)	C. 12-18 min	C vs A (p)	D. 18-24 min	D vs A (p)	E. 24-30 min	E vs A (p)
95% Area (mm^2^)	647.71 ± 262.53	634.25 ± 258.33	NS	559.88 ± 215.55	0.006	523.34 ± 186.49	0.002	550.14 ± 208.64	0.003
X Sway Amplitude (mm)	6.06 ± 1.22	5.89 ± 1.36	NS	5.45 ± 0.94	0.016	5.4 ± 0.89	0.010	5.37 ± 1.04	0.000
Y Sway Amplitude (mm)	7.51 ± 2.03	7.7 ± 1.91	NS	7.22 ± 1.64	NS	6.77 ± 1.61	0.016	7.12 ± 1.67	NS
Path Length (mm)	2486.95 ± 468.62	2473.77 ± 494.62	NS	2260.03 ± 443.96	0.009	2153.48 ± 315.35	0.002	2215.4 ± 371.13	0.009
LFS (mm^-1^)	4.54 ± 1.92	4.63 ± 2.05	NS	4.67 ± 2.04	NS	4.67 ± 1.77	NS	4.54 ± 1.39	NS
Velocity (mm/s)	41.45 ± 7.81	41.23 ± 8.24	NS	37.67 ± 7.4	0.009	35.89 ± 5.26	0.002	36.92 ± 6.19	0.009

P values refer to comparison of each time point with the initial one (0–6 min). NS: Not Significant.

It is noteworthy that the training-induced changes in unipedal stance parameters were correlated with their initial values (time 6 min). When pooled changes at times 18–24 and 24–30 were taken in into account, significant, negative correlations with initial values could be observed for 95% Area (R = 0.668, Y = −0.359X + 121.831, p < 0.0005), Y (R = 0.587, Y = −0.365X + 2.173, p < 0.0005) and X (R = 0.610, Y = 0.369X + 1.562, p < 0.0005) Sway Amplitude, Path Length (R = 0.680, Y = −0.516X + 981.877, p < 0.0005) and Velocity (R = 0.680, Y = −0.516X + 16.36, p < 0.0005). The same result was also found for LFS (R = 0.566, Y = −0.282X + 1.342, p = 0.001), although its average value did not change significantly during training.

**Fig 4:**
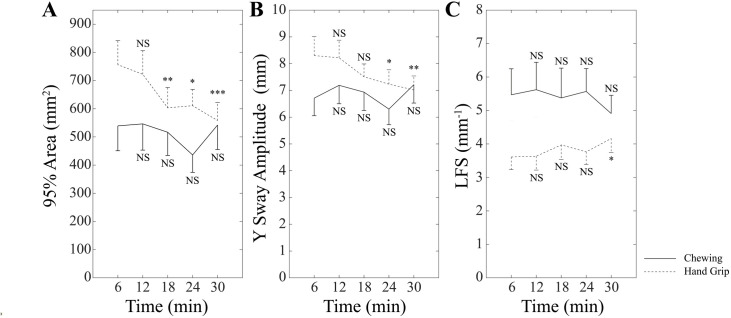
Between-group differences in 95% Area, Y Sway Amplitude and LFS during postural training. The values of 95% Area (A), Y Sway Amplitude (B) and LFS (C) unipedal stance values obtained in the Chewing (continuous lines) and Hand Grip (dotted lines) groups have been plotted as a function of the time lapsed from the beginning of postural training. Error bars represent SE. Each time point has been compared to the initial (0-6 min) one. NS: not significant. *: p < 0.05. **: p < 0.01. ***: p < 0.005.

No significant Group effects were present, while significant Time x Group effects were observed for the 95% Area (F(4,56)=3.97, p = 0.007), Y Sway Amplitude (F(4,56)=3.41, p = 0.015) and LFS (F(4,56)=2.74, p = 0.038) (Fig 4).

This analysis disclosed that 95% Area and Y Sway Amplitude decreased progressively and significantly in the HG group, in contrast to the C group. The LFS values significantly increased at the end of the training period only in the HG group (p = 0.044). 95% Area and Y Sway Amplitude of HG and C groups were not significantly different, whatever time point was considered. LFS values were significantly higher in the C with respect to the HG group at time points 0–6 (p = 0.049), 6–12 (p = 0.048) and 18–24 minutes (p = 0.036). When these parameters were separately evaluated for males and females, it appeared that, in both C and HG groups, no significant sex-related differences could be observed by unpaired t test whatever the timepoint considered. Moreover, parameter changes showed similar trends as a function of time in males and females.

No significant between-group differences were observed in the tiredness score, thus indicating that the subjective fatigue perception was comparable in the two groups. During the 30 min period of postural training, falls could be occasionally observed in few subjects. No significant Time, Group effects and interaction could be observed when the frequency of falls was analysed by a 5 Time x 2 Group repeated measures ANOVA.

**Fig 5:**
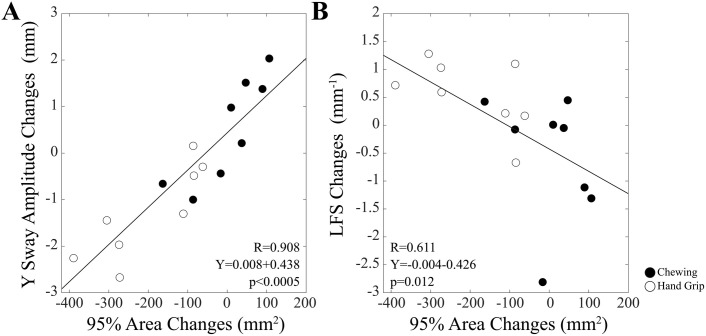
Relation of the changes in Y Sway Amplitude and LFS induced by postural training with those in 95% Area. The changes induced by postural training in Y Sway Amplitude (A) and LFS (B) have been plotted as a function of the corresponding changes in 95% Sway Area. Subjects of the Chewing and Hand Grip groups are indicated by filled and open symbols, respectively. The continuous lines correspond to the regression lines calculated for all the plotted points, whose data have been reported in the panels.

It is noteworthy that changes at time 24−30 min (with respect to initial values) of Y Sway Amplitude and LFS were strongly correlated with 95% Area changes (Fig 5). A correlation was also observed between LFS and Y Sway Amplitude changes (R = 0.570, Y = −0.365X-0.032, p < 0.0005). These findings held true for both C and HG groups. Surprisingly, changes in LFS were independent upon those in Path Length in the whole population (R = 0.022, p = 0.904), as well as in the individual groups.

**Fig 6:**
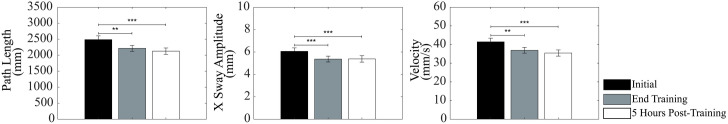
Consolidation of training-induced changes in unipedal stance: Path Length, X Sway Amplitude and Velocity. The values of Path Length (A), X Sway Amplitude (B) and Velocity (C) obtained for pooled subjects of the Chewing and Hand Grip groups before (black bar), at the end (grey bar) and 5 hours post-training (white bar) have been reported. Error bars represent SE. The second and third time points have been compared to the initial one. Only significant differences have been reported. *: p < 0.05. **: p < 0.01. ***: p < 0.005.

**Fig 7:**
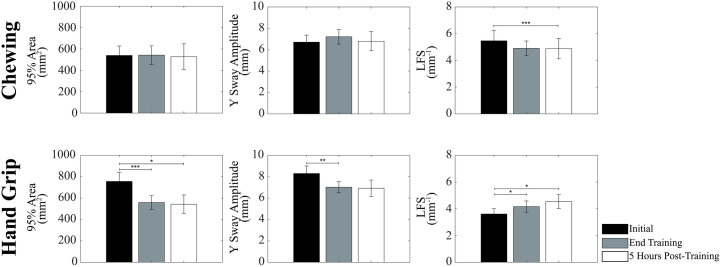
Consolidation of training-induced changes in unipedal stance: 95% Area, Y Sway Amplitude and LFS. The values of 95% Area (A), Y Sway Amplitude (B) and LFS (C) obtained for subjects of the Chewing (upper row) and Hand Grip (lower row) groups before (black bar), at the end (grey bar) and 5 hours post-training (white bar) have been reported. Error bars represent SE. The second and third time points have been compared to the initial one. Only significant differences have been reported. *: p < 0.05. **: p < 0.01. ***: p < 0.005.

In general, the training-induced changes in unipedal stance parameters were persistent, since the values observed at the end of the training period were close to those observed 5 hours later. This held true for both C and HG groups taken separately, as well as for the whole population (Figs 6 and 7).

### Training-induced changes in bipedal stance

The effects of unipedal training on bipedal stance parameters were separately investigated for each eye and support condition by a 3 Time repeated measures ANOVA with Group as between-subjects factor.

Significant/quasi significant Time x Group interactions were observed exclusively in the OE-SS condition for 95% Area (F(2,28)=9.16, p = 0.001), X Sway Amplitude (F(2,28)=4.092, p = 0.045), LFS (F(2,28)=6.103, p = 0.006), Velocity (F(2,28)=3.729, p = 0.05) and Path Length (F(2,28)=3.729, p = 0.05), which are decomposed in Table 2.

**Table 2:** Mean ± SD of the different parameters relative to bipedal stance on soft support with the eyes open recorded in the Chewing and Hand Grip groups.

		A. Baseline	A vs B (p)	B. End Training	B vs C (p)	C. Five Hours Post-Training	C vs A (p)
95% Area (mm^2^)	Chewing	391.54 ± 184.56	NS	470.39 ± 227.44	NS	555.31 ± 264.62	0.010
	Chewing vs Hand Grip (p)	NS		NS		NS	
	Hand Grip	428.8 ± 138.31	0.011	313.45 ± 127.05	NS	355.24 ± 199.51	NS
X Sway Amplitude (mm)	Chewing	4.17 ± 1.01	NS	5.01 ± 2.10	NS	5.22 ± 1.30	0.004
	Chewing vs Hand Grip (p)	NS		NS		NS	
	Hand Grip	4.79 ± 1.08	NS	4.21 ± 0.87	NS	4.27 ± 1.32	NS
Y Sway Amplitude (mm)	Chewing	6.54 ± 2.00	NS	7.05 ± 0.59	NS	7.95 ± 3.13	NS
	Chewing vs Hand Grip (p)	NS		0.008		NS	
	Hand Grip	6.24 ± 1.47	NS	5.27 ± 1.40	NS	5.51 ± 1.86	NS
Path Length (mm)	Chewing	1087.26 ± 145.06	NS	1107.6 ± 181.98	NS	915.59 ± 153.54	NS
	Chewing vs Hand Grip (p)	NS		NS		NS	
	Hand Grip	1184.7 ± 173.51	0.001	976.73 ± 157.54	NS	1029.79 ± 178.37	0.047
LFS (mm^-1^)	Chewing	3.18 ± 1.06	NS	2.69 ± 0.88	0.006	1.9 ± 0.69	0.001
	Chewing vs Hand Grip (p)	NS		NS		0.016	
	Hand Grip	3.11 ± 1.32	NS	3.45 ± 1.09	NS	3.63 ± 1.64	NS
Velocity (mm/s)	Chewing	18.12 ± 2.42	NS	18.46 ± 3.03	NS	15.26 ± 2.56	NS
	Chewing vs Hand Grip (p)	NS		NS		NS	
	Hand Grip	19.74 ± 2.89	0.001	16.28 ± 2.63	NS	17.16 ± 2.97	0.047

Data have been acquired before (Baseline, A), soon after (End Training, B) and 5 Hours Post-Training (C). NS: Not Significant.

The significant changes occurring in the parameters reflecting the range of CoP excursion (95% Area, X Sway Amplitude) were of opposite signs in C (increases) and in HG (drops) groups. On the other hand, Velocity (and Path Length) values were significantly lowered following the training period only in the HG group. Consistently, LFS significantly decreased in C, but not in the HG group. When the behaviour of males and females was analysed, it appeared that between group differences in the OE-SS condition were present only in the C group, for 95% Area (females: 279.17 ± 94.49 mm^2^, males: 130.04 ± 39.47 mm^2^, p = 0.027) and X Sway Amplitude (females. 4.13 ± 1.12 mm, males: 2.30 ± 0.46 mm, p = 0.039) evaluated at the beginning of the session. Moreover, the changes observed between the different timepoints were closely corresponding in the two sexes.

**Fig 8:**
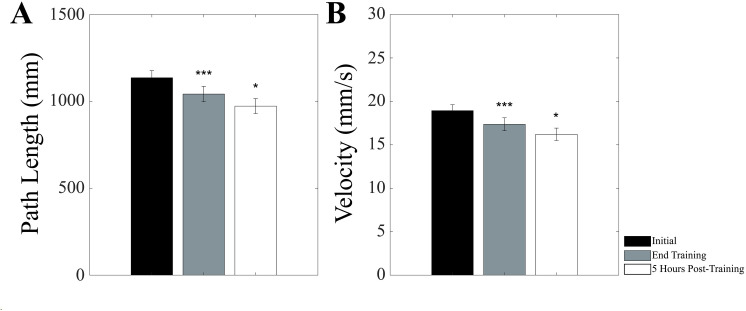
Training-induced changes in bipedal stance. The values of Path Length (A) and Velocity (B) obtained in bipedal stance with eyes closed on a soft support for pooled subjects of the Chewing and Hand Grip groups before (black bar), at the end (grey bar) and 5 hours post-training (white bar) have been reported. Error bars represent SE. The second and third time points have been compared to the initial one. Only significant differences have been reported. *: p < 0.05. **: p < 0.01. ***: p < 0.005.

A Time effect could be observed in OE-SS condition for Velocity (F(2,28)=5.342, p = 0.018) and Path Length (F(2,28)=5.342, p = 0.018), due to a significant decrease of post-training and 5 hour Velocity values compared to control, which is shown in Fig 8.

**Fig 9:**
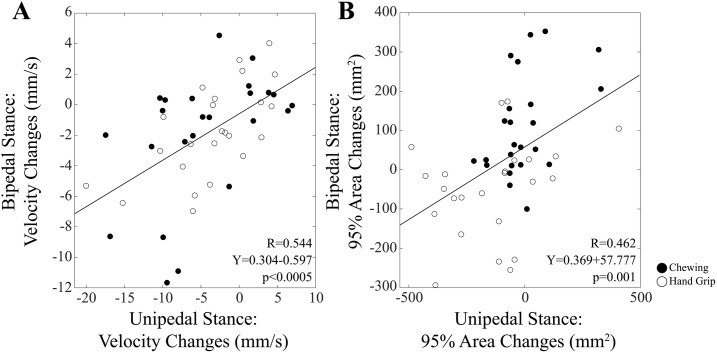
Relation between training-related changes in unipedal and bipedal stance. The relation between the training induced changes in unipedal and bipedal (eyes open, soft support) stances have been plotted for Velocity (A) and 95% Area (B). Subjects of the Chewing and Hand Grip are indicated by filled and open symbols, respectively. The continuous lines correspond to the regression lines for all the plotted points, whose data have been reported in the panels.

To study whether the observed changes in bipedal stance could be effectively related to unipedal stance training, pooled changes (post training-control, 5 hour-control, 5 hour-post training) obtained for bipedal stance parameters were correlated with the corresponding changes in unipedal stance. A significant correlation was observed for Velocity (Fig 9A) and Path Length (R = 0.544, Y = 0.304X-35.804, p < 0.0005) (not shown).

In addition to Path Length and Velocity changes, also changes in 95% Area (Fig 9B) and LFS (R = 0.405, Y = 0.460X-0.307, p = 0.004) showed a significant correlation between OE-SS bipedal and unipedal stance values. No corresponding correlation was observed for X Sway Amplitude.

### Effects of motor activities and stability of postural parameters

The acute effect of chewing and hand grip on unipedal stance parameters was assessed by comparing the first and the second unipedal stance record in the control session (Fig 2), where these activities were performed between the first and the second recording in unipedal stance. This goal was achieved by a 2 Recording (first, second) repeated measures ANOVA with Group (C, HG) as a between-subjects factor. This analysis did not reveal significant Recording effect and interaction, whatever parameter considered, indicating lack of acute effect of the motor activity on the unipedal stance. This outcome was confirmed when a 2 Recording repeated measures ANOVA was separately performed for the two groups.

The stability of postural control was investigated by correlating for each parameter the values observed in the experimental and in the control session. Independently upon the Group, when subjects stood in unipedal stance on a hard support, eyes open, strong correlations between sessions were found for all CoP parameters, except for the X Sway Amplitude (Table 3). In bipedal stance, no correlation was observed for X Sway Amplitude and Velocity, whatever the eye and the support condition. Significant between session correlations were found for both 95% Area and LFS, in all the open eyes conditions of bipedal stance, and for Y Sway Amplitude in the OE-HS and in the CE-SS condition.

**Table 3:** Correlations between unipedal and bipedal stance parameters recorded at the beginning of the training and of the control session.

		Unipedal Stance	OE-HS	CE-HS	OE-SS	CE-SS
95% area (mm^2^)	R	0.778	0.716	0.234	0.793	0.117
	p	0.001	0.019	NS	0.001	NS
	Slope	0.727	0.803		1.123	
X Sway Amplitude (mm)	R	0.432	0.277	0.236	0.467	0.032
	p	NS	NS	NS	NS	NS
	Slope					
Y Sway Amplitude (mm)	R	0.785	0.663	0.159	0.523	0.598
	p	0.001	0.010	NS	NS	0.031
	Slope	0.76	0.729			0.568
LFS (mm^-1^)	R	0.572	0.564	0.524	0.793	0.202
	p	0.033	0.036	NS	0.001	NS
	Slope	0.405	0.351		1.083	
Velocity (mm/s)	R	0.851	0.174	0.079	0.301	0.490
	p	<0.0005	NS	NS	NS	NS
	Slope	0.918				

Values of coefficients of correlation (R), slope and probability (p) of the null hypothesis (lack of correlation) are reported. OE: Open Eyes. CE: Closed Eyes. SS: Soft Support. HS: Hard Support. NS: Not Significant.

### Masseter EMG asymmetry, anisocoria and postural parameters

The EMG activity during clenching was studied in 8 subjects of the C and in 6 of the HG group. Within these populations a strong EMG asymmetry during clenching was observed (42.3 ± 19.9%, range: 24.4–93.8%), without significant between-group differences; 3 subjects (in HG group) showed a right and 11 a left dominance. No correlation was found between the EMG asymmetry and the angle of rotation observed in the Fukuda test, irrespectively of using relative or absolute asymmetry values. The EMG asymmetry, evaluated as a relative or absolute value was not correlated with the postural parameters of bipedal (whatever condition was considered) and unipedal stance. Moreover, it did not influence the changes in the unipedal stance parameters observed following the training.

Most of the subjects showed an anisocoria when the arches were kept slightly apart, whose average, absolute value corresponded to 0.33 ± 0.29%. No correlation was found between EMG and pupil asymmetry, irrespectively whether relative or absolute values were considered. Bipedal and unipedal stance parameters, as well as training-induced changes in unipedal stance, were independent upon both relative and absolute values of anisocoria. Similar results were obtained when the anisocoria values taken with the arches in contact were considered.

## Discussion

### Pre-training activity and postural learning: Reliability of Time x Group effects

Consistently with previous investigations [60], the results of the present experiments show that the indicators of unipedal stance stability were improved when this posture was repeated 10 times at 2–3 minute intervals. When all the subjects were considered together, time related changes of unipedal postural parameters during training were homogeneous: 95% Area, X and Y Sway Amplitude, Velocity decreased progressively for 24 min, then levelled or slightly raised in the last 6 min of training (time 30 min). On the other hand, no significant Time effect could be observed for LFS. As previously shown, the training induced change observed for each parameter was negatively correlated with the initial value.

The present findings indicated that the conditioning motor activity, which does not modify unipedal stance acutely, as indicated by the results of the control session, can influence the training effects. While in HG group a decrement in 95% Area and Y Sway Amplitude was observed during the training, with a parallel increase in LFS, these parameters were not significantly modified in the C group. Strong correlations between the changes in these parameters were observed in both groups, indicating that the different motor activities performed before the beginning of the training sequence favoured different time evolutions of CoP parameters.

### Chewing and postural learning

Due to the lack of a group of subjects performing the training period without preceding motor activities, the present experiments do not allow to assess the effect of chewing on postural learning per se, but only in comparison to a different conditioning activity. The present study documents a lack of difference in CoP Velocity and X Sway Amplitude time course during training between the C and the HG groups. It must be pointed out that the overall decrease in CoP Velocity and X Sway Amplitude is like that observed in a previous study, where no conditioning motor activity preceded the training period [60]. Thus, chewing does not exert on postural control the same positive effect that has been documented on cognitive performance. Such an effect is not replicated by an handgrip exercise [7,12].

This observation was somehow surprising, given the strong trigeminal input to the LC. Because of trigeminal activation of LC, an increase in the norepinephrine release is expected to occur at the level of the cerebellum [68,69], where the noradrenergic input facilitates the plastic changes affecting the postural reflexes [66,67]. Thus, based on previous studies [60], we would have expected a greater training-induced postural stability in unipedal stance within the C with respect to the HG group.

Considering that 95% Area and Y Sway Amplitude were not significantly modified by the training session in the C and decreased in the HG group, at a first glance, handgrip might be considered more efficient than chewing in stimulating postural learning.

A lack of chewing effect on postural training could be explained assuming that trigeminal input to the LC does not affect the cerebellar projecting neurons. There is, at present, no experimental evidence for supporting this hypothesis. Initially, the central noradrenergic system was considered a non-specific system whose neurons projected to the whole brain [78], but more recent evidence indicated that different population of LC neurons may be related to different projections and neural functions [79]. Thus, it is possible that specific afferent inputs such as those arising from the trigeminal region control selected populations of neurons. Some degree of segregation of the afferent inputs to the LC neurons has been indeed documented: the core region of the LC, where the cell bodies are located, is targeted only by signals arising from the prepositus hipoglossi, the paragigantocellularis reticular nucleus [80] and the orexin neurons of the lateral hypothalamus [81], while other afferent systems reach the peripheral pericoerulear region where dendrites of LC cells are located [80]. In this respect, trigeminal afferent input is in part carried out through the praepositus hypoglossi and the paragigantocellularis regions [43,82]. Further studies are needed to assess the possible lack of trigeminal input to LC neurons projecting to the cerebellum.

Since the decrease in 95% Area and Y Sway Amplitude, which can considered as the expression of a higher postural stability [83], are not observed following a conditioning period of chewing, it could be concluded that chewing is detrimental for postural learning. This hypothesis, however, is at variance with the positive effects of the noradrenergic system on postural learning which has been documented in animal experiments [66,67].

However, another possibility can be considered. Analysis of CoP fluctuations suggests that CoP motion is regulated by (sensory) feedback and feedforward mechanisms which act on relatively longer and shorter timescale, respectively. The feedforward mechanism could arise from a descending command to postural muscles [84]. Thus, to at least in principle, a larger CoP sway area could be due to a stronger feedforward process, rather than to a poor feedback control. Within such a frame, populations of athletes well trained in peculiar sports may display larger CoP fluctuation at rest with respect to non-trained or less trained subjects of comparable age, while their CoP velocity is lower [85]: in these instances, postural skill could consist in the ability to maintain balance being compliant with CoP oscillations. This postural strategy can be observed also in subjects showing a peculiar cognitive tract, i.e., a high susceptibility to be hypnotized. It has been shown, in fact, that these individuals show a larger feedforward displacement of CoP with respect to subjects characterized by a low susceptibility [86], while feedback processes are similar. Yet, both groups show the same capability to maintain balance in front of dynamic perturbations [87].

We may hypothesize that chewing induces an increase in the release of norepinephrine at cerebellar level, which does not modify the adaptation process enhancing the reduction of CoP Velocity, but interferes with the postural strategy of the subject, favouring the maintenance of a large surface of CoP excursion. This form of adaptation may contribute to maintain stable LFS values, that is a stable level of energy expenditure associated to postural control [88]. In this respect, it of interest that larger LFS values have been shown to occur when attentional physical and cognitive performance deteriorates [89], a condition which is likely associated to a lower enhancement of LC activity during tasks [90].

### Learning transfer from unipedal to bipedal stance

When all the subjects were pooled together, postural learning induced changes not only in unipedal, but also in bipedal stance Path Length and Velocity, which were reduced immediately and 5 hours after training. Changes in unipedal and bipedal stance were correlated to each other, suggesting a transfer of learning from the trained (unipedal) to the untrained (bipedal) posture. This finding was at variance with previous investigations performed in the absence of a conditioning motor activity, showing that the postural improvement in unipedal stance transferred to bipedal stance only the day after the training (and just regarding the 95% Area). Prolonged periods of exercise in challenging conditions have given conflicting results, either absence [91,92] or presence of transfer to bipedal stance [93]. The earlier skill transfer observed in the present study could be therefore attributed to the conditioning motor activity that preceded the postural training.

In this study following a single session of postural training the transfer was substantially limited to the OE-SS condition, which bears two similarities with the training condition:

1. an instability of the stance, due to the foam that makes difficult the use of proprioceptive information for postural stabilization [86], and
2. a continuous flow of visual information that helps postural stabilization and that is experienced by the subject also during the training period.

Comparison of the C and HG groups revealed that a reduction of CoP Path Length and Velocity in bipedal stance was significant only for the latter group. This finding could be tentatively attributed to a higher specificity of the learning process in the C group, as far as the control of CoP Velocity is concerned. Within the frame of a chewing-induced stimulation of the noradrenergic system, this finding could appear contradictory, since an increase in the tonic LC activity due to a stressing condition enhances (aversive) learning generalization [94]. However, pupil size recordings indicate that chewing enhances the task-related phasic activity of LC neurons, rather than their basal discharge [7]. Moreover, the effects of tonic and task-related activation of LC neurons on neural circuits controlling cognitive performance are opposite to each other [95], an increase in phasic activity being associated to an increased cognitive performance [90]. So, the possibility that the increased specificity of the learning process observed in the C group is due to a chewing-induced enhancement in the phasic release of norepinephrine at cerebellar level during postural training cannot be excluded.

Post-training between-group comparison also enlighten, in the C group, an increase of bipedal stance 95% Area and a decrease of LFS values that were not observed in unipedal stance. Although the changes in unipedal stance following training were not significant, they correlated with the (significant) changes in bipedal stance, supporting the training-dependent nature of the latter. So, within the C group, postural learning modified postural control in bipedal stance, favouring a larger compliance to CoP excursion and a lower energetic cost of balance. It is possible that this change in postural strategy was achieved by an enhanced release of norepinephrine at cerebellar level.

### Stability of postural parameters

Comparison of postural data recorded in sessions separated by at least 6 weeks showed several correlations between the two datasets, indicating that individual subjects have some tendency to adopt a stable postural strategy, except for the control of lateral sway. In bipedal stance, this phenomenon was observed in the eyes open condition (5/8 correlations being significant, see Table 3), rather than in the closed eyes condition (1/8 correlation being significant). In unipedal stance (eyes open), all parameters but X Sway Amplitude showed significant correlations between sessions. These findings indicated that the primary factor favouring a stability of postural strategy is the availability of vision. The second factor is likely to be adopting a less stable posture, such as unipedal standing.

### Limitations of the study

The present report presents limitations that must be overcome in future studies. First, the presence of a side dominance for chewing was not evaluated and related to the handedness of the subjects. Moreover, the chewing rate was not externally paced (e.g., via metronome), nor were spatial or temporal aspects of mastication reported. So, estimation of the rhythmic sensorimotor input hypothesized to drive the observed effects is lacking. Finally, no direct neurophysiological outcomes or assessments, that could have further validated the proposed mechanisms, were included in the study.

### Conclusions

The results of the present experiments indicate that the time course of changes in CoP Velocity and Path Length observed during postural training are similar when the training is conditioned by a short period of chewing or handgrip activities. However, only handgrip promotes a generalization of such a skill to bipedal stance. On the other hand, the evolution of 95% Area, which does not decrease during training in the C group, suggests that chewing promotes a postural strategy characterized by a higher compliance to the extent of CoP displacement and a lower energy cost which is transferred to bipedal stance, where the sway area is significantly increased following postural training. Further experiments are necessary to verify to what extent such changes may promote a more secure balance and what are the effects of repeated training sessions over week/month periods.
